# *Citrus aurantium* L. and Its Flavonoids Regulate TNBS-Induced Inflammatory Bowel Disease through Anti-Inflammation and Suppressing Isolated Jejunum Contraction

**DOI:** 10.3390/ijms19103057

**Published:** 2018-10-07

**Authors:** Wei He, Yongmin Li, Mengyang Liu, Haiyang Yu, Qian Chen, Yue Chen, Jingya Ruan, Zhijuan Ding, Yi Zhang, Tao Wang

**Affiliations:** 1 Institute of Traditional Chinese Medicine, Tianjin University of Traditional Chinese Medicine, Tianjin 300193, China; WeiHe850227@gmail.com (W.H.); liumengyang0212@tjutcm.edu.cn (M.L.); qianchen6688@gmail.com (Q.C.); YueChen17208@gmail.com (Y.C.); 2 College of Traditional Chinese Medicine, Hebei North University, Zhangjiakou 075000, China; yongminli1020@gmail.com; 3 Tianjin Key Laboratory of TCM Chemistry and Analysis, Institute of Traditional Chinese Medicine, Tianjin University of Traditional Chinese Medicine, Tianjin 300193, China; hyyu@tjutcm.edu.cn (H.Y.); ruanjingya123@gmail.com (J.R.); zding2772@gmail.com (Z.D.)

**Keywords:** *Citrus aurantium* L., naringenin, nobiletin, hesperetin, inflammatory, jejunum contraction

## Abstract

Inflammatory bowel disease (IBD) is a serious digestive system disease, for which the clinical therapeutic choices remain limited. Dried fruits of *Citrus aurantium* L. (CAL) are a traditional medicine used for regulation of the digestive system. The aim of this study was to identify the regulatory effects of CAL on IBD and to clarify the mechanism of the active compounds. In trinitrobenzene sulfonic acid-induced IBD rats, 125 to 500 mg/kg of oral CAL significantly alleviated weight loss and diarrhea, decreased colitis inflammatory cell infiltration, and inhibited pro-inflammatory cytokine production. The mechanisms of characteristic flavonoids in CAL were evaluated involving inflammation and intestine contraction aspects. Naringenin, nobiletin, and hesperetin showed anti-inflammatory effects on lipopolysaccharide-induced RAW cells. The mechanism may be related to the inhibition of the tumor necrosis factor-α (TNF-α)-induced nuclear factor kappa-light-chain-enhancer of activated B cells (NF-κB) pathway to suppress cyclooxygenase-2 (COX-2) and inducible nitric oxide synthase (iNOS) expressions. Naringenin and nobiletin showed inhibitory effects on isolated jejunum contraction. The mechanism of naringenin is partly related to COX, NOS, inositol triphosphate (IP_3_), and finally, to decreased jejunum motility. This study demonstrated that CAL, and its flavonoids’ regulatory effects on IBD through anti-inflammation and inhibition of intestine muscle contraction, can provide basic information on developing new drugs or supplements against IBD based on CAL.

## 1. Introduction

Inflammatory bowel disease (IBD) is a serious digestive system disease, characterized as a chronic and relapsing inflammation of the gastrointestinal tract [1]. Epidemiological studies showed a large variation in the prevalence of IBD in different regions in the world. The highest incidence is noted in the United States of America (USA), where approximately 2.5 million inhabitants have IBD, ranging from the age of six to over 60 [2,3,4]. Although IBD is a rare disease in Asia, population-based studies reported the annual incidence of IBD increased fivefold from 1990 to 2016 with a continuous long-term increasing tendency [5,6,7]. The rising prevalence is likely to become a substantial challenge to Asian countries.

Patients with IBD may have symptoms of abdominal cramping, bloody diarrhea, and bloating with a change in stool frequency and form. These symptoms are embarrassing and uncomfortable, and they seriously affect the quality of life. Gut luminal factors, such as food, microbiota, and bile acids, together with their internal interactions might be important for the generation of symptoms in IBD patients. The etiology of IBD is related to various factors, including physical factors (diet, medication, and natural course of illness), psychological factors (anxiety, depression, trauma, and loss), and social factors (financial, vocational, and residential). The pathogenesis of IBD is yet to be fully elucidated [8].

Pathological characteristics of IBD include a reduction in mucus layer thickness, inflammatory cell infiltration in the tunica mucosa and tunica submucosa, an ablated mucosa layer, the presence of ulcers in the muscular layer, a dropout of crypt epithelial cells, and the destruction of epithelia and lamina propria in the intestine lumen.

Until now, there is no appropriate drug for IBD treatment. Clinically, some medication was used to relieve symptoms. Salicylazosulfapyridine was used to relieve IBD inflammation [9]. Azathoprine, an immunosuppressive medication, was used for the inhibition of the immune response [10]. However, the above drugs are not enough for clinical IBD therapeutic requirements.

The fruit of *Citrus aurantium* L. (CAL), commonly named bitter orange, a member of genus *Citrus* (Rutaceae), is widely used as an edible and medicinal resource in China. CAL contains essential oils (limonene, linalool, and citral), flavonoids (naringenin, hesperetin, and apigenin), triterpenoids (limonin and limonexin), coumarins (marmin, meranzin, and scopoletin), and alkaloids (synephrine and *N*-methyltyramine) [11]. Previous studies demonstrated that CAL extracts and its main components had anti-cancer, anti-inflammatory, anti-oxidant, and vasodilatory effects [12,13,14,15,16].

In traditional Chinese medicine (TCM), CAL is used as a qi regulatory component in digestive formula. Our research group reported the effects of CAL on gastric emptying and gastro-protection [17]. As a further study, in this paper, we found that CAL 70% ethanol (EtOH) extract had an amelioratory effect on 2,4,6-trinitrobenzene sulfonic acid (TNBS)-induced IBD rats. The molecular underlying mechanisms of anti-inflammation and jejunum contraction inhibition were elucidated, which indicated that CAL may be beneficial for treating IBD.

## 2. Results

### 2.1. CAL Improves Symptoms of TNBS-Induced IBD Colitis in Rats

**Figure 1:**
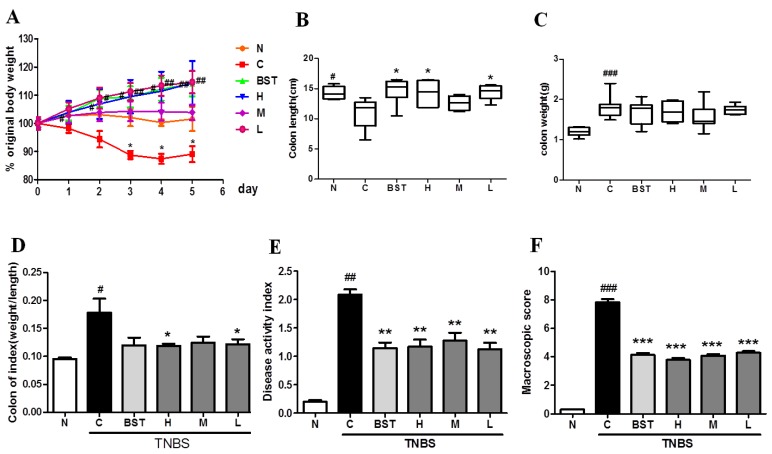
*Citrus aurantium* L. (CAL) alleviates 2,4,6-trinitrobenzene sulfonic acid (TNBS)-induced colitis in rats. The time-course of changes on day 4 after TNBS-induced inflammatory bowel disease (IBD) in (**A**) body weight; (**B**) colon length; (**C**) colon weight; (**D**) the ratio of weight/length; (**E**) disease activity index (DAI) score; and (**F**) macroscopic injury score. N: normal group; C: control (TNBS only) group; BST: balsalazide 1 g/kg + TNBS group; H (high): CAL 500 mg/kg + TNBS group; M (medium): CAL 250 mg/kg + TNBS group; L (low): CAL 125 mg/kg + TNBS group; *n* = 8, # *p* < 0.05, ## *p* < 0.01, ### *p* < 0.001 vs. normal group; * *p* < 0.05, ** *p* < 0.01, *** *p* < 0.001 vs*.* control group.

To define whether CAL had a positive effect on TNBS-induced rats, a series of indices were measured in the experiment, such as body weight, diarrhea and bloody-stool incidence, colon length, etc. In this study, compared to the normal group, significant reductions in body weight (Figure 1A) and colon length (Figure 1B), along with obvious increases in colon weight (Figure 1C), the ratio of weight/length (Figure 1D), disease activity index (DAI; Figure 1E), and macroscopic injury score (Figure 1F), were found in the TNBS-treated group. Furthermore, the results showed that CAL alleviated TNBS-induced weight loss, diarrhea, and bloody stool, and significantly reduced DAI and macroscopic injury score. Moreover, we found that pre-treatment with CAL significantly alleviated TNBS-induced colon shortening, and decreased colon weight and the ratio of weight/length. Taken together, these results indicated that CAL reduced TNBS-induced acute colitis.

**Figure 2:**
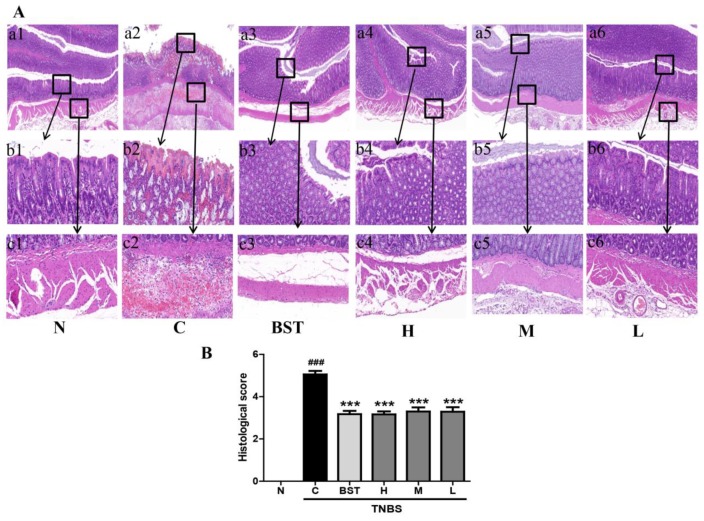
CAL suppresses histological injury in TNBS-induced colitis in rats. (**A**) Representative images of hematoxylin and eosin (H&E) staining of colon tissue from different groups (a1–a6). Scale bar = 100 μm. The area within the rectangle in each picture is enlarged and presented below, correspondingly, displaying the mucosa (b1–b6) and submucosa (c1–c6) in each group. Scale bar = 10 μm. (**B**) Colonic histological score. N: normal group; C: TNBS only group; BST: balsalazide 1 g/kg + TNBS group; H: CAL 500 mg/kg + TNBS group; M: CAL 250 mg/kg + TNBS group; L: CAL 125 mg/kg + TNBS group; *n* = 8, ### *p* < 0.001 vs. normal group; *** *p* < 0.001 vs*.* TNBS group. Data (mean ± standard error of the mean (SEM)) were analyzed by ANOVA.

Next, we measured the effects of CAL on colorectal histology in rats with TNBS-induced colitis. As shown in Figure 2A, unlike the normal group, TNBS treatment caused serious inflammation with a scattered infiltration of monocytes. However, CAL treatment demonstrated a lower level of inflammation with scattered infiltration of monocytes in TNBS-treated rats and a decrease in the morphological alteration. This result indicated CAL has protective effects on intestinal cytoarchitecture in colonic layers and inhibitory effects on inflammation (Figure 2B). 

### 2.2. CAL Suppresses Inflammation in TNBS-Induced Colitis

**Figure 3:**
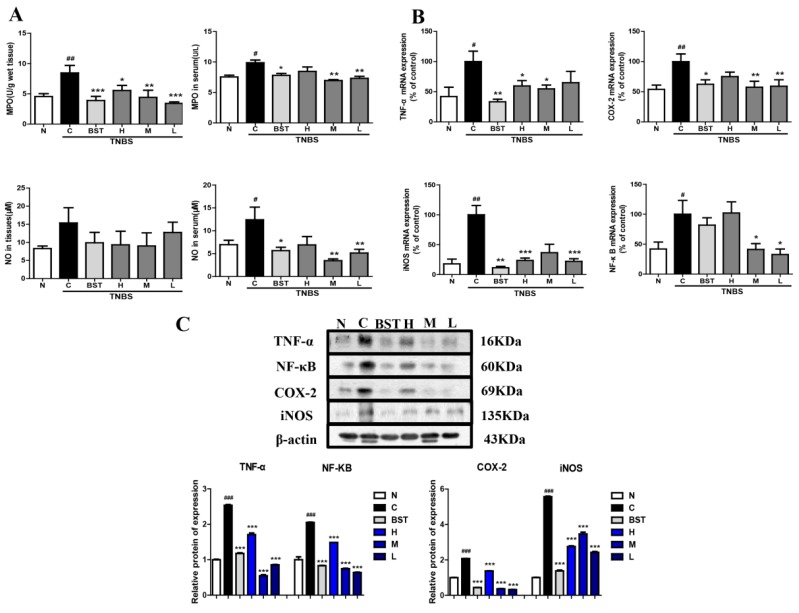
Inhibitory effects of CAL on the pro-inflammatory cytokine production of TNBS-induced IBD rats. (**A**) Nitric oxide (NO) and myeloperoxidase (MPO) content in serum and colon tissue. (**B**) The mRNA expressions of pro-inflammatory cytokines in colon tissue. (**C**) The protein expressions of pro-inflammatory cytokines in colon tissue. N: normal group; C: TNBS alone group; BST: balsalazide 1 g/kg + TNBS group; H: CAL 500 mg/kg + TNBS group; M: CAL 250 mg/kg + TNBS group; L: CAL 125 mg/kg + TNBS group; *n* = 8, # *p* < 0.05, ## *p* < 0.01, ### *p* < 0.001 vs. normal group; * *p* < 0.05, ** *p* < 0.01, *** *p* < 0.001 vs. TNBS group. Data (mean ± SEM) were analyzed by ANOVA.

An increase in myeloperoxidase (MPO) and nitric oxide (NO) levels represents an aggravated inflammatory reaction. In order to clarify the anti-inflammatory effects of CAL, the levels of MPO and NO were detected. As shown in Figure 3A, compared with the normal group, there was an obvious increase in the model group. CAL treatment significantly inhibited TNBS-induced MPO activity in both colon tissues and sera. Furthermore, CAL dramatically decreased TNBS-stimulated NO production. Tumor necrosis factor-α (TNF-α), cyclooxygenase-2 (COX-2), inducible nitric oxide synthase (iNOS), and nuclear factor kappa-light-chain-enhancer of activated B cells (NF-κB) are the major inflammatory cytokines in IBD. The RT-PCR and Western blot results show that TNBS significantly induced pro-inflammatory cytokine messenger RNA (mRNA) and protein levels of TNF-α, COX-2, iNOS, and NF-κB in colon tissues, while CAL downregulated their expressions in different levels (Figure 3B,C). 

### 2.3. The Effects of CAL, Naringenin, Nobiletin, and Hesperetin on Mouse-Isolated Jejunum Contractility

Although hesperidin and naringine are the major flavonoids in CAL, as literature reports, after oral administration, both of these flavonoids are enzymatically hydrolyzed by intestinal microbiota, yielding their aglycons, hesperetin and naringenin, respectively [18]. Nobiletin is a characteristic citrus flavonoid with a polymethoxylated structure, whose structure is resistant to modification by intestinal microbiota [19].

**Figure 4:**
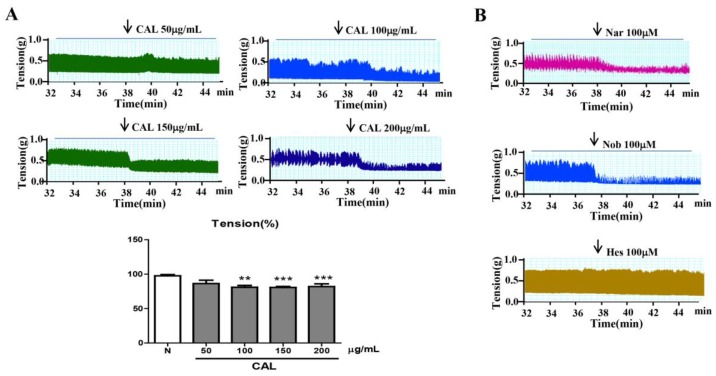
Effect of CAL, naringenin, nobiletin, and hesperetin on isolated jejunum contraction. (**A**) Effect of CAL extraction on isolated jejunum contraction. (**B**) Effects of naringenin, nobiletin, and hesperetin on isolated jejunum contraction. Nar: naringenin; Nob: nobiletin; Hes: hesperetin; *n* = 6, ** *p* < 0.01, *** *p* < 0.001 vs. normal group. Data (mean ± SEM) were analyzed by ANOVA.

To clarify the amelioratory effect of CAL on TNBS-induced colitis, firstly, we investigated the effects of CAL and hesperetin, naringenin, and nobiletin on mouse jejunum contractility. As shown in Figure 4, CAL extraction (from 100–200 µg/mL), as well as naringenin and nobiletin (100 µM), dramatically inhibited the active tension on spontaneous contractions of intestine smooth muscle. However, at the same concentration, hesperetin showed no significant changes on isolated jejunum movement.

### 2.4. Mechanism Studies of Naringenin and Nobiletin on Jejunum Contractility

**Figure 5:**
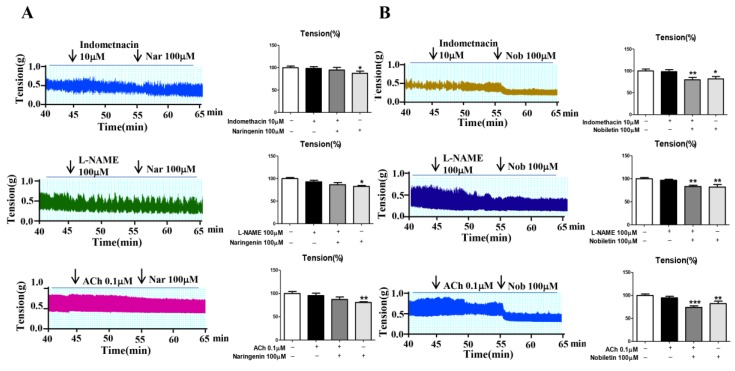
Inhibition mechanisms of (**A**) naringenin and (**B**) nobiletin on isolated jejunum contraction. Nar: naringenin; Nob: nobiletin; *n* = 6, * *p* < 0.05, ** *p* < 0.01, *** *p* < 0.001 vs. normal group. Data (mean ± SEM) were analyzed by ANOVA.

Indomethacin (blocker of prostaglandin I2 (PGI2)), N(ω)-nitro-l-arginine methyl ester (L-NAME; the inhibitor of NOS), acetylcholine (ACh; activator of inositol triphosphate (IP3), was used to clarify the mechanism of flavonoids of CAL on jejunum contractility, As shown in Figure 5, indomethacin (10µM), L-NAME (100µM) and Ach (0.1µM) could completely antagonist the inhibition effect of naringenin on mouse jejunum contractility, but have no significant effect on nobiletin. 

### 2.5. Anti-Inflammatory Effect of Naringenin, Nobiletin, and Hesperetin in Lipopolysaccharide (LPS)-Induced RAW264.7 Cells

**Figure 6:**
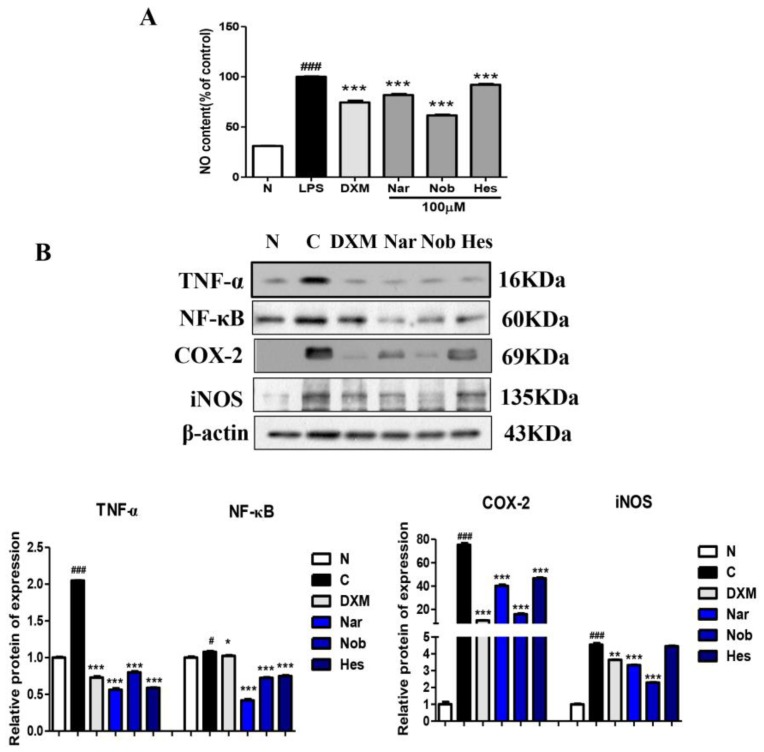
Inhibitory effects of naringenin, nobiletin, and hesperetin on pro-inflammatory cytokine production in lipopolysaccharide (LPS)-induced RAW264.7 cells. (**A**) NO content in the supernatant of RAW264.7 cells. (**B**) The protein expressions of pro-inflammatory cytokines in LPS-induced RAW264.7 cells. N: normal group; C: LPS only group (0.5 µg/mL); DXM: dexamethasone 1 µg/mL + LPS (0.5 µg/mL); Nar: naringenin (100 µM) + LPS (0.5 µg/mL); Nob: nobiletin (100 µM) + LPS (0.5 µg/mL); Hes: hesperetin (100 µM) + LPS (0.5 µg/mL); *n* = 6, # *p* < 0.05, ### *p* < 0.001 vs. normal group; * *p* < 0.05, ** *p* < 0.01, *** *p* < 0.001 vs. LPS group. Data (mean ± SEM) were analyzed by ANOVA.

We also investigated whether naringenin, nobiletin, and hesperetin inhibited LPS-induced pro-inflammatory cytokines. As shown in Figure 6A, compared with the normal group, LPS caused a significant increase in NO production in Raw264.7 cells, while naringenin, nobiletin, and hesperetin treatment dramatically inhibit LPS-stimulated NO production. Furthermore, compared to the normal group, LPS led an obvious upregulation in the protein expressions of TNF-α, NF-κB, COX-2, and iNOS. Naringenin, nobiletin, and hesperetin also strongly inhibited the protein expressions of TNF-α, NF-κB, COX-2, and iNOS in LPS-induced RAW264.7 cells (Figure 6B). Taken together, our results suggest that naringenin, nobiletin, and hesperetin have beneficial anti-inflammatory effects in LPS-induced RAW264.7 cells.

## 3. Discussion

IBD is a gastrointestinal tract disease, which is characterized by chronic, relapsing inflammation and abnormal intestinal contraction. Current medications can help treat IBD, but direct therapeutic medicine for IBD does not yet exist. In this paper, we firstly reported that CAL, a kind of fruit, had regulatory effects on IBD. We partially clarified the mechanism of CAL’s major flavonoids, naringenin and nobiletin, on the suppression of inflammation and regulation of jejunum motility.

In the present study, pre-treatment with CAL in a TNBS-induced colitis model showed an improvement in weight loss, diarrhea, and bloody stool, ameliorated colon weight, colon DAI, and macroscopic scores, and increased colon length and decreased the ratio of weight/length. This result indicated that CAL showed benefits for symptomatic relief in IBD abdominal discomfort. Intestine spasm is the key factor in abdominal pain and diarrhea or loose stool. Previous studies demonstrated that decreased intestinal contractility led to a reduction in gastrointestinal motility, which in turn alleviated abdominal pain and diarrhea in IBD patients [20,21]. CAL significantly reduced isolated jejunum contraction, revealing the potential therapeutic use in the treatment of IBD abdominal discomfort.

Overloading inflammatory response is a basic pathological process through the occurrence and development of IBD [22,23,24]. Colon tissue pathological examination results showed that CAL treatment significantly improved the intact mucosal layer and intestinal glands, and decreased the infiltration and erosion of inflammatory cells. The inflammation suppressing effect of CAL was further confirmed by a reduction in NO and MPO levels in both serum and colon tissue. The mechanism may be related to the TNF-α-induced NF-κB activation pathway for reducing the expressions of COX-2 and iNOS.

Naringenin, nobiletin, and hesperetin are the characteristic flavonoids in CAL [11,25]. In this paper, preliminary screening showed that naringenin and nobiletin had stronger inhibitory effects on isolated jejunum contraction, but hesperetin had no significant effect. From a chemical structure perspective, the only difference between naringenin and hesperetin is the hydroxy substitution in the B cycle, which is 4′-hydroxy in naringenin and 3′-hydroxy-4′-methoxy in hesperetin. This result indicated that 4′-hydroxy is the essential group for inhibitory effects on jejunum contraction; however, evaluations involving numbers should be carried out to validate the structure–activity relationship.

In IBD patients, the intestinal tract motility index and high-amplitude propagating contractions were significantly greater than in healthy volunteers. Abnormal intestinal tract contraction is one of the causes of abdominal pain or discomfort. In smooth-muscle contraction, calcium triggers a contraction via a reaction with regulatory factors. The mechanism for jejunum contraction is carried out via the release of calcium ions. The inhibitory effect of naringenin was blocked by indomethacin (the inhibitor of COX, blocks the generation of PGI2, leading to an increase in intracellular Ca^2+^, and has an anti-relaxation effect on smooth muscle) [26], L-NAME (the inhibitor of NOS, increases intracellular Ca^2+^, leading to an anti-relaxation effect) [27], ACh (activates IP_3_), causes increased intracellular Ca^2+^, and promotes the contractile activity of the intestinal smooth muscle) [28], indicating that the effect is at least partly related to COX, NOS, and IP_3_, and finally, the decrease in intracellular Ca^2+^. However, there were no significant changes between nobiletin with or without the above inhibitors; thus, the mechanism needs to be further explored.

According to the anti-inflammatory effects, naringenin, nobiletin, and hesperetin showed important protective roles on LPS-induced RAW264.7 cells, and significantly decreased the level of NO in the cell supernatant. The mechanism was related to the inhibition of the TNF-α-induced NF-κB pathway responsible for suppressing the expressions of COX-2 and iNOS, in accordance with CAL extract in TNBS-induced rats.

In summary, on the basis of in vitro and in vivo studies, our study demonstrated that CAL, as an edible fruit, and its flavonoids showed significant regulatory effects on TNBS-induced IBD rats through anti-inflammation and the inhibition of jejunum muscle contraction. These results provide molecular information for further investigation of the mechanisms via which CAL moderates IBD. The intestine barrier is a key factor in IBD, and the contribution of CAL to mucosal barrier function needs further investigation.

## 4. Materials and Methods

### 4.1. Materials

CAL was provided by Tianjin Zhongxin Pharmaceutical Group Co., Ltd. (Tianjin, China), for which the content of Naringin was 3.8%, as determined using HPLC/ultraviolet (UV) detection [29]. Naringenin (purity >98%, HPLC), nobiletin (purity >98%, HPLC), and hesperetin (purity >98%, HPLC) were purchased from Shanghai Yuanye Bio-Technology Co., Ltd. (Shanghai, China). Balsalazide (H20041706) was purchased from Shanxi Zhendong Ante Biopharmaceutical Co., Ltd. (Shanxi, China). Cell culture reagents and supplies were purchased from Hyclone Laboratories, Inc. (Logan, UT, USA). Rabbit anti-TNF-α, COX-2, NF-κB, iNOS, and β-actin were purchased from Abcam plc. (Cambridge, MA, USA). Horseradish peroxidase-conjugated anti-rabbit immunoglobulin G (IgG) were purchased from Zhongshan Goldbridge Biotechnology (Beijing, China). TNBS, ACh, L-NAME, indomethacin, dimethyl sulfoxide (DMSO), dexamethasone, and LPS were obtained from Sigma Chemical Co., (St. Louis, MO, USA). NO and MPO detection kits were purchased from Nanjing Jiancheng Bioengineering Institute (Nanjing, China).

### 4.2. Animals

Healthy male adult Sprague/Dawley (SD) rats weighting 180–200 g were received from Beijing HFK Bioscience Co. Ltd. (Beijing, China; Certificate of Conformity: No. 11401300071030). All animal experiments were approved by the Science and Technological Committee and the Animal Use and Care Committee of TUTCM (No. 201712003). Animals were housed in cages in a room with controlled temperature (22 ± 2 °C), relative humidity (40–60%), and a 12-h light/dark cycle throughout the study. The animals were acclimated to their environment for one week and had ad libitum access to tap water and a rodent standard diet (crude protein 16%, crude fat 4%, crude fiber 12%, and ash 8%).

### 4.3. TNBS-Induced Experimental Colitis

After one week of adaption, 48 rats were randomly divided into six groups, each consisting of eight rats. The groups were as follows: normal group (N), control group (C), positive control group (PC), and CAL groups. CAL was suspended in 5% acacia (Sigma-Aldrich Inc., St. Louis, MO, USA) solution, and oral administration volumes were 10 mL/kg body weight; the final doses were 500, 250, and 125 mg/kg. The normal and control groups received 5% acacia water solution with the same volume, while the positive control group received balsalazide 5% acacia water solution. The same treatments were conducted once every day for three consecutive days.

Experimental colitis was induced according to previously established protocols with slight modifications [30,31]. On the fourth day, rats were fasted for 24 h and they had free access to drinking water. On the fifth day, rats were anesthetized with diethyl ether and a catheter was inserted through the anus so that its terminus reached approximately to the level of the splenic flexure (8 cm proximal to the anal verge). Subsequently, 1 mL of TNBS dissolved in ethanol (50% *v*/*v*) was infused at a dose of 100 mg/kg. Throughout the TNBS challenge period, all groups received the same treatment as the pretreatment. During the experiment, body movement, body weight, diarrhea incidence, and bloody stool were recorded daily.

### 4.4. Colon Damage Assay

On the ninth day, the blood of rats was collected from orbit, and then, rats were sacrificed under ether anesthesia by cervical dislocation for the assessment of colon damage. The colon length and weight were measured. The colon macroscopically visible damage was scored based on the literature-reported method [32,33] (Table 1). DAI score was determined as previously reported [34] (Table 2). Routine hematoxylin and eosin (H&E)-stained colon sections according to previously described morphological criteria and the damages were both assessed blindly by two investigators according to a modified histological grading scale, which takes both inflammatory cell infiltration and tissue damage into consideration [35] (Table 3).

### 4.5. Detection of Inflammatory Cytokines and Mediators in Colon and Serum

Colon samples (40–50 mg) were homogenized with saline (1:9 *w*/*v*) in a digital homogenizer. After centrifugation at 3500× *g* for 10 min, the supernatant was collected for further detection. Blood samples were centrifuged at 3500× *g* for 10 min, and the serum was transferred into new Eppendorf tubes. All the above experiments were kept under 10 °C.

The levels of NO and MPO in both serum and colon tissue were measured using commercial kits. Inflammatory-related cytokines and mediators such as COX-2, iNOS, TNF-α, and NF-κB were analyzed using the methods of RT-PCR and Western blot as described below.

### 4.6. Effects of CAL and Its Flavonoids on Mice Jejunum Contraction

After fasting for 24 h, mice were sacrificed, and about 1 cm of the jejunum was cut down. The preparations were mounted longitudinally to an isometric force transducer with a silk-braided non-absorbable suture (Johnson & Johnson Medical China Ltd., Beijing, China), and were allowed to equilibrate in an organ bath with 10 mL of Tyrode’s buffer (1 L contains NaCl 8.0 g, CaCl_2_ 0.2 g, KCl 0.2 g, MgCl_2_ 0.1 g, NaHCO_3_ 1.0 g, KH_2_PO_4_ 0.05 g, and glucose 1.0 g; pH 7.4) for 30 min to achieve a stable state. The organ bath was maintained at a constant temperature (37.0 ± 0.5 °C), and bubbled with 95% O_2_ and 5% CO_2_ gas. Intestine contractions were recorded using the Power Lab system and the Chart 7 software (AD instrument Ltd., New South Wales, Australia). Indomethacin (10 µM), L-NAME (100 µM), and Ach (0.1 µM) were used for mechanism researches.

### 4.7. Inflammatory Response Induced by LPS on RAW264.7

RAW264.7 cells were obtained from the cell center at the Chinese Academy of Medical Science and Peking Union Medical College (Beijing, China). The RAW264.7cells were cultured in Dulbecco’s modified Eagle medium (DMEM), supplemented with 10% fetal bovine serum (FBS), penicillin (100 units/mL), streptomycin (100 mg/mL), l-glutamine (4.5 mg/mL), and glucose (4.5 mg/mL), and incubated at 37 °C in a humidified atmosphere containing 5% CO_2_ and 95% air. The media were refreshed every two days.

Initially, 2 × 10^6^ cells/mL RAW264.7 cells were seeded on a 24-well plate and incubated overnight. The next day, the media were changed, which contained LPS (0.5 µg/mL) with or without naringenin (100 µM), hesperetin (100 µM), nobiletin (100 µM), and the positive drug dexamethasone (1 µg/mL), and then, the cells were incubated for 24 h. The cell supernatant was collected to detect NO levels, and cells were harvested for protein and RT-PCR analysis.

### 4.8. RT-PCR Analysis

Total RNA was extracted from colon tissues and RAW264.7 cells using TRIzol reagent (Sigma, USA) and complementary DNA (cDNA) was generated using RT-PCR reagent (Thermo Fisher Sci. Inc., Vilnius, Lithuania). RT-PCR was performed using the SYBR Green Quantity Tech RT-PCR kit (Thermo Fisher Sci. Inc., St. Austin, TX, USA) through PCR 7500. The housekeeping gene, glyceraldehyde 3-phosphate dehydrogenase GAPDH), served as an internal control. The comparative *C*t method (2^−△△^*^C^*^t^) was used to analyze differences in the levels of detective mRNA between groups. The sequences of the primers used in this investigation are shown in Table 4.

### 4.9. Western Blot Analysis

As described previously [36], the rats’ colon segments and the RAW264.7 cells treated with LPS were analyzed by Western blot. The protein concentrations in the supernatants and tissues were quantified using a bicinchoninic acid protein assay kit (Thermo Fisher Sci. Inc., Rockford, IL, USA). Firstly, 60 µg of protein was mixed with 4× loading dye (Laemmli Buffer) and 2-mercapto ethanol, before being heated at 95 °C for 5 min. The protein was resolved by 8–12% sodium dodecyl sulfate polyacrylamide gel electrophoresis and transferred to immunoblot polyvinylidene difluoride (PVDF) membranes (Merck Millipore Ltd., Darmstadt, Germany). The membranes were incubated at 4 °C overnight with primary antibodies against TNF-α (1:1000) (ab6671, Abcam), COX-2 (1:1000) (ab52237, Abcam), NF-κB (1:1000) (ab16502, Abcam), iNOS (1:800) (ab3523, Abcam), and β-actin (1:1000) (ab8227, Abcam). Then, the membranes were washed three times with Tris-buffered saline/Tween 20 (TBS-T; 10 min each time) and incubated with a horseradish peroxidase-labeled secondary goat anti-rabbit (1:10,000) antibody for 1 h at room temperature. Next, the blots were again washed three times with TBS-T (10 min each time). 

Finally, protein bands were visualized with an enhanced chemiluminescence system (Millipore, Billerica, MA, USA). The relative optical densities of protein bands were analyzed with the Amersham imager 600 luminescent image analyzer (GE healthcare Japan Co., Tokyo, Japan).

### 4.10. Statistic Analysis

Values were expressed as means ± SD. All the grouped data were statistically analyzed with the SPSS 11.0 software. Significant differences between the normal group or control group were evaluated by one-way analysis of variance (ANOVA), and Tukey’s studentized range test was used for post hoc evaluations. A *p*-value <0.05 was considered to indicate statistical significance.

## Figures and Tables

**Table 1:** Evaluation of macroscopic scores.

Colon Damage	Score
No damage	0
Hyperemia with ulcers	1
Hyperemia and wall thickening without ulcers	2
One ulceration site without wall thickening	3
Two or more ulceration sites	4
0.5-cm extension of inflammation or major damage	5
1-cm extension of inflammation or severe damage	6–10

**Table 2:** Evaluation of disease activity index (DAI) scores.

DAI Score	Weight Loss (%)	Stool Consistency	Occult/gross Bleeding
0	None	None	None
1	1–5	Loose	Hem occult positive
2	5–10		
3	10–15	Diarrhea	Gross bleeding
4	>15		

**Table 3:** Evaluation of histological scores.

Inflammatory Cell Infiltration		Tissue Damage	
No infiltration	0	No mucosal damage	0
Increased number of inflammatory cells in the lamina propria	1	Discrete epithelial lesions	1
Inflammatory cells extending into the submucosa	2	Erosions or focal ulcerations	2
Transmural inflammatory cell infiltration	3	Severe mucosal damage with extensive ulceration extending into the bowel wall	3

**Table 4:** Primers used for RT-PCR analysis. COX-2—cyclooxygenase 2; TNF-α—tumor necrosis factor alpha; iNOS—inducible nitric oxide synthase; NF-κB—nuclear factor kappa-light-chain-enhancer of activated B cells; GAPDH—glyceraldehyde 3-phosphate dehydrogenase; F—forward; R—reverse.

Species	Gene	Primer Sequence	
*Rat*	*COX-2*	F: TCGGAGGAGAAGTGGGTTTTAG	R: TTGATGGTGGCTGTCTTGGTAGG
	*TNF-α*	F: GATGTGGAACTGGCAGAGGAG	R: CACGAGCAGGAATGAGAAGAG
	*iNOS*	F: TTGGAGCGAGTTGTGGATTGTT	R: TAGGTGAGGGCTTGCCTGAGTG
	*NF-* *κ* *B*	F: AACACTGCCGACCTCAAGAT	R: CATCGGCTTGAGAAAAGGAG
	*GAPDH*	F: TGAGGCCGGTGCTGAGTATGT	R: CAGTCTTCTGGGTGGCAGTGA

## Abbreviations

IBD	inflammatory bowel disease
TNBS	2,4,6-trinitrobenzene sulfonic acid
NO	nitric oxide
MPO	myeloperoxidase
H&E	hematoxylin and eosin
DAI	disease activity index
TNF-α	tumor necrosis factor-α
COX-2	cyclooxygenase-2
iNOS	inducible nitric oxide synthase
NF-κB	nuclear factor kappa-light-chain-enhancer of activated B cells
LPS	lipopolysaccharide
ACh	acetylcholine
L-NAME	N(ω)-nitro-l-arginine methylester hydrochloride
NOS	nitric oxide synthase
IP_3_	inositol triphosphate
